# Quality of Life and Healthcare Experiences of Patients with Anal Cancer: A Mixed-Methods Study

**DOI:** 10.3390/curroncol33050266

**Published:** 2026-05-05

**Authors:** Andreia F. Moura, Catarina S. Padilla, Samuel M. Vorbach, Emily I. Holthuis, Baukelien van Triest, Cristiane D. Bergerot, Vassilios Vassiliou, Emir Celik, Kübra Akkaya, Irfan Cicin, Winette T. A. van der Graaf, Olga Husson, Samantha C. Sodergren

**Affiliations:** 1 Quality of Life Department, European Organisation for Research and Treatment of Cancer (EORTC) Headquarters, 1200 Brussels, Belgium; 2 Department of Medical Oncology, The Netherlands Cancer Institute, 1066 CX Amsterdam, The Netherlands; c.simoes@nki.nl (C.S.P.); e.holthuis@nki.nl (E.I.H.); w.vd.graaf@nki.nl (W.T.A.v.d.G.); o.husson@nki.nl (O.H.); 3 Department of Medical Oncology, Erasmus MC Cancer Institute, University Medical Centre Rotterdam, 3015 GD Rotterdam, The Netherlands; 4 Department of Public Health, Erasmus MC, University Medical Centre Rotterdam, 3015 GD Rotterdam, The Netherlands; 5 Department of Radiation Oncology, Medical University of Innsbruck, 6020 Innsbruck, Austria; samuel.vorbach@i-med.ac.at; 6 Department of Radiation Oncology, The Netherlands Cancer Institute, 1066 CX Amsterdam, The Netherlands; b.v.triest@nki.nl; 7 Department of Supportive Care, Oncoclinicas&Co—Medica Scientia Innovation Research (MEDSIR), Sao Paulo 13571-410, SP, Brazil; cristiane.bergerot@oncoclinicas.com; 8 Department of Radiation Oncology, Bank of Cyprus Oncology Centre, Nicosia 2006, Cyprus; vasilis.vasiliou@bococ.org.cy; 9 Department of Medical Oncology, Faculty of Medicine, Istinye University, Istanbul 34396, Turkey; emir.celik@isu.edu.tr (E.C.); irfan.cicin@istinye.edu.tr (I.C.); 10 Department of Medical Oncology, Prof. Dr. Cemil Taşcıoğlu City Hospital, Istanbul 34000, Turkey; drkubraakkaya@gmail.com; 11 Department of Surgical Oncology, Erasmus MC Cancer Institute, University Medical Centre Rotterdam, 3015 GD Rotterdam, The Netherlands; 12 School of Health Sciences, University of Southampton, Southamptom SO17 1BJ, UK; s.c.sodergren@soton.ac.uk

**Keywords:** anal cancer, rare cancers, health-related quality of life, healthcare experience, EORTC, qualitative interviews, mixed-methods

## Abstract

Anal cancer is a rare and under-researched malignancy, leading to limited understanding of patients’ experiences and potentially insufficiently tailored care. This study explored the health-related quality of life (HRQoL) and healthcare interactions of people with anal cancer. Patients with confirmed diagnosis took part in semi-structured interviews, supplemented by two European Organisation for Research and Treatment of Cancer (EORTC) HRQoL questionnaires. Data were analysed using Interpretative Phenomenological Analysis and organized into themes. Twenty-one patients (71% female; mean age of 62 years) from five countries were included. HRQoL challenges were identified across four phases: illness onset, diagnosis, treatment, and life beyond treatment. Key themes included misdiagnosis, not being taken seriously, and emotional and social disruptions. Additional themes involved stigma, embarrassment, strain on loved ones, and healthcare experiences. Defecation problems were especially burdensome, beginning at onset, intensifying during treatment, and persisting as a chronic issue affecting well-being. Patients described coping strategies and sometimes reframed their experiences positively, expressing gratitude for support received. Questionnaire findings aligned with patients’ reports of prominent physical symptoms. Anal cancer remains highly stigmatized, creating complex physical, emotional, and social challenges. Individualized care and extended psychosocial and practical support beyond treatment are essential to improve HRQoL and dignity in survivorship.

## Simple Summary

Anal cancer is a rare illness, and little is known about how it affects people’s everyday lives. This study explored the experiences of 21 patients from five countries to better understand their quality of life and interactions with healthcare. Patients took part in interviews and completed two questionnaires about symptoms and well-being. Many patients reported delays in diagnosis, often feeling dismissed or not taken seriously. Shock, fear, and emotional strain were common, as were practical challenges such as severe pain, bowel problems, and soreness, which limited daily and social activities. Bowel problems often began before diagnosis, worsened during treatment, and in many cases continued long after treatment ended. Although most patients described positive interactions with healthcare providers, especially during treatment, follow-up care felt less supportive. Despite difficulties, many patients developed positive coping strategies. The findings highlight the need for more ongoing emotional and practical support beyond treatment.

## 1. Introduction

Anal cancer is a rare malignancy, accounting for less than 1% of all cancers in the UK and 0.5% in the US, and it ranked 30th globally across all cancer sites [1,2,3]. Historically, anal cancer has been under-researched compared to other gastrointestinal cancers and has often fallen under the umbrella of colorectal cancer in terms of consensus recommendations, clinical trial design and supportive care [4]. While rectal cancer often involves surgical resection with (neo)adjuvant therapy, localised anal cancer is primarily treated with intensive concurrent radical chemoradiotherapy. These differences result in unique side effect profiles and survivorship challenges, underscoring the need for separate research and tailored supportive care approaches [5]. While prognosis is favourable for patients treated for localised anal cancer (5-year survival ranges between 80 and 85%), the aggressive treatment protocols are associated with high rates of severe toxicity (e.g., to the skin, bowel, urinary tract, and sexual organs), significantly impacting health-related quality of life (HRQoL) [5,6].

HRQoL examines the patient’s perception of how their diagnosis and treatment may impact their physical, social, and psychological well-being daily [7]. The first published systematic review of the HRQoL issues experienced by patients with anal cancer included only 11 studies [6,8,9,10,11]. None of the studies adopted a qualitative design, and therefore, the HRQoL issues reported were a function of the questions included in the non-anal-cancer-specific questionnaires. A subsequent review published in 2019 included only 15 studies [12], while a recent review of HRQoL and measures used for solid rare cancers extracted 17 studies evaluating HRQoL of patients with anal cancer, showing a modest but continuous progression and interest in research for this population [13]. The European Organisation for Research and Treatment of Cancer (EORTC) developed a HRQoL questionnaire specific to anal cancer patients managed with radical chemoradiotherapy: the EORTC QLQ-ANL27 [6,14,15]. As part of its development, interviews were carried out with 43 patients with anal cancer to generate a comprehensive list of issues [14]. However, no in-depth qualitative analysis was undertaken, limiting the depth of knowledge. Importantly, while HRQoL instruments capture predefined domains, they may not fully reflect the broader lived experience of anal cancer, including psychosocial, relational, and identity-related impacts that emerge across the disease trajectory.

Recent studies on anal cancer embedding qualitative methods have typically been limited in scope. Approaches have included adding a free-text question to Patient Reported Outcome (PRO) measures to capture experiences in the patients’ own words and conducting semi-structured interviews with small samples from a single setting [16]. For example, one study interviewed only four patients with anal cancer (alongside other rare cancer types) and 12 health care professionals (HCPs), and another combined the results of 14 interviews from patients with colorectal and anal cancer (*n* = 3) [17]. Lee Mortensen et al. acknowledged the need for larger samples and that including only six patients in their study limited their ability to confirm their findings and explore any age and gender differences [18]. Collectively, these studies highlight both the feasibility and value of qualitative approaches in anal cancer research, while underscoring persistent limitations related to sample size, disease-specific focus, and depth of analysis.

The scarcity of research on anal cancer has significant implications for clinical practice and survivorship care. At the start of their disease trajectory, patients commonly face significant diagnostic delays [13]. These delays often stem from low clinical suspicion, symptom overlap with benign conditions (e.g., haemorrhoids), and limited awareness among healthcare professionals, leading to a lack of appropriate examinations [19], often compounded by stigma, embarrassment, and the sensitive nature of anorectal symptoms. Evidence shows that the average time from symptom onset to diagnosis for anal cancer can exceed seven months [20,21]. Furthermore, limited evidence and poor understanding of the unique challenges faced by this population mean that healthcare delivery is often sub-optimal and insufficiently tailored to their needs. Patients with anal cancer have critiqued the amount of information provided and support offered for managing late effects of treatment [16].

To date, no qualitative study has comprehensively explored the lived and healthcare experiences of patients with anal cancer across countries and stages of the disease trajectory. Extending previous research, the current study adopts a more comprehensive approach and is part of a wider project examining healthcare experiences and HRQoL of patients with solid rare tumours [22]. The current study aims to answer two main questions: (1) What are the lived experiences of patients with anal cancer? (2) What is this patient group’s experience with the healthcare system? Following phenomenological traditions, we define ‘lived experience’ as the subjective, first-person meanings individuals attribute to events as they are encountered in everyday life [23]. We aim to draw on insights from multiple care settings across different countries and across different stages of the disease and treatment trajectory.

## 2. Methods

### 2.1. Setting, Population, and Data Collection

This is an explanatory mixed-methods study, drawing on qualitative interviews and quantitative questionnaires. Quantitative data from questionnaires were included as a triangulation technique to complement the in-depth qualitative analysis from interviews [24]. In this explanatory design, the qualitative component constitutes the primary source of inquiry, with quantitative data serving a complementary role to contextualize and further explain the qualitative findings. This triangulation approach enhances the validity of the findings by allowing the strengths of each method to compensate for the limitations of the other [24]. 

The data presented here describes a predefined subgroup analysis of the larger multicentre EORTC QoL Rare Cancer study (003-2022)—which aimed to develop a HRQoL measurement strategy for patients with solid rare cancers [22]. For this study, only patients with a confirmed anal cancer diagnosis aged ≥18 years were included. Participants were introduced and invited to the study by their clinical team, following a convenience sampling strategy, meaning that recruitment depended on the accessibility and availability of patients at the time.

Given that this analysis involved secondary use of data collected within the primary study protocol, the sample size (as well as the distribution across cultures, languages, and stages of the treatment trajectory) was determined by the design and recruitment processes of the parent study rather than by criteria established specifically for the present research. Consequently, it was not possible to define or balance the sample size per cultural or linguistic group a priori. In line with qualitative research principles, particularly within an interpretivist–phenomenological paradigm, the emphasis of this study is placed on the contextual detail and depth of each participant’s account rather than on proportional representation.

Sociodemographic information was self-reported by the patient or obtained from electronic health records (EHRs) and confirmed by the institution’s medical team. It included variables such as marital status, education and employment status, and travel distance and time to the hospital. Clinical characteristics included time since diagnosis, type of disease, presence and number of metastases, treatment intent and type, comorbidities, and Eastern Cooperative Oncology Group Performance Status (ECOG) scores. For a full explanation of the recruitment process, see Padilla et al. 2023 [22].

This study was conducted according to the Declaration of Helsinki. Ethical approval was obtained from the Institutional Review Board of The Netherlands Cancer Institute (IRBd22-298/17 January 2023) and by local ethical committees and national regulatory authorities from each participating centre.

### 2.2. Semi-Structured Interviews

The interview schedule included the administration of questionnaires (EORTC QLQ-C30 and the EORTC QLQ-ANL27), followed by questions about the understanding and relevance of these questionnaires and health-related aspects throughout the illness trajectory. The present study focuses on the results of the open-ended questions on HRQoL and experiences with health care services, for example: “Can you give me a summary about your experiences surrounding the rare cancer from the time you started experiencing symptoms, the time cancer was diagnosed and then the treatment(s) you received up to and including now?” and “Have you experienced any problems as a result of your rare cancer diagnosis? If so, what problems have you experienced?”

Interviews were conducted either by a researcher not involved in the participants’ direct clinical care (The Netherlands, England) or by a doctor working in the local clinic (Austria, Turkey, Brazil) in person or by telephone. Most were conducted in English (*n* = 7), which were recorded and transcribed verbatim. The interviews conducted originally in another language (i.e., Dutch, Arabic, Portuguese, German) had an English transcription or summary (*n* = 14), including field notes, provided to the study coordinator (C.S.P.). To minimise the risk of misinterpreting data collected in languages other than English, native or fluent-speaking authors reviewed the original interview materials (including transcriptions and field notes in the source language) to verify the accuracy of the translated content provided to the study coordinator.

Field notes, which capture participants’ verbal reports, are a well-established method in qualitative research [25,26]. The use of verbal reports draws methodological inspiration from ethnographic research, where field notes serve as the central unit of analysis. Such notes may include direct quotations but often also reflect the researchers’ interpretations and observations [26]. In this study, field notes were recorded by professionals with expertise in both clinical practice and qualitative interviewing. These notes captured details such as body language and other culturally relevant cues that could influence how patients discussed their illness.

### 2.3. Questionnaire Application

HRQoL was assessed using EORTC questionnaires: the EORTC QLQ-C30 [10] and the EORTC QLQ-ANL27 [14].

The EORTC QLQ-C30 is a validated generic core cancer questionnaire [10], translated into more than 100 languages and used internationally in oncology research. It is composed of 30 items and includes five functioning scales, three symptom scales, a global health status scale, and six single items.

The EORTC QLQ-ANL27 was designed to supplement the EORTC QLQ-C30 with the inclusion of anal-cancer-specific questions [14]. The questionnaire includes 27 questions covering five domains: bowel symptoms (non-stoma), bowel symptoms (stoma), stoma care, pain/discomfort, vaginal symptoms, and nine single items (frequent urination, keeping clean, proximity to toilet, lower limb oedema, planning activities, sexual interest, sex life, painful sexual intercourse, and erectile problems) [14].

Patients were asked to complete the two questionnaires based on relevance on a 4-point Likert scale (1—not relevant at all; 2—somewhat relevant; 3—relevant; 4—very relevant). Relevance ratings were used to explore the perceived importance of HRQoL issues from the patient perspective rather than symptom severity alone. Subsequently, participants were asked to indicate the ten most relevant questions from both questionnaires combined. All questionnaires were presented in the participants’ native or preferred language. Although the EORTC QLQ-ANL27 contains conditional instructions directing specific items to be completed only by patients with a stoma bag (items 47–49), men (item 54), or women (items 55–57), all items were administered to all participants as per the EORTC Rare Cancer QoL project protocol. In the project’s framework, the primary objective is to evaluate the perceived relevance of each item across the full patient population, irrespective of applicability under standard module instructions. Participants were therefore instructed to respond to all items and to select ‘1 = not relevant at all’ if an item was not applicable to them.

### 2.4. Analysis

Descriptive statistics (i.e., percentage of missing data, mean, standard deviation) for sociodemographic and clinical data were analysed. Questionnaire data included descriptive statistics, proportion of respondents rating the item as relevant, response ranges, and priority rating (frequency of the selected items in the “top ten”). The mean cut-off in questions was >1.5, and ceiling and floor effects were determined if >80% participants scored “very relevant (4)” or “not relevant at all (1)”, according to the EORTC module development guidelines [27,28]. All analyses were conducted in SPSS (version 30.0.0).

Patient interview transcripts and field notes were exported into NVIVO (version 15) to support and structure the analysis. Reflexive thematic analysis was used as an analytic method, informed by the epistemological and interpretative principles of Interpretative Phenomenological Analysis (IPA) [29,30]. The analysis was grounded in IPA, a methodological approach that prioritizes an in-depth, interpretive examination of how individuals make sense of their lived experiences [23]. Accordingly, this study was situated within an interpretivist–phenomenological research paradigm [23]. Three authors (C.S.P., S.C.S., and A.F.M.), with expertise in qualitative methods, led the data analysis and met regularly throughout the coding process to discuss and refine the interpretation of identified themes. Additional authors involved in data collection (S.M.V., E.I.H., and K.A.) reviewed the coding framework and contributed to writing the Results Section. To manage potential variability arising from mixed data sources, we employed a systematic and iterative analytic process. All materials (i.e., verbatim transcripts, translated summaries, and field notes) were coded within the same analytic framework. Regular team discussions helped ensure interpretive consistency across countries and data types.

## 3. Results

### 3.1. Social Demographic and Clinical Characteristics

Twenty-one patients with an anal cancer diagnosis were included. Patients had a mean age of 61.9 years (SD = 9.3), and most were female (71.4%). Most patients were from the United Kingdom (33.3%), followed by Austria and The Netherlands (28.6% each). The mean time since diagnosis was 17.6 months (SD = 17.4), and most patients had localised disease (90.5%). Treatment was received or ongoing with curative intent (90.5%), and most patients received, at some point, chemotherapy (71.4%), radiotherapy (71.4%), or concurrent chemoradiotherapy (33.3%). Full descriptive data are presented in Table 1.

### 3.2. Interviews

#### 3.2.1. Thematic Analysis

The interviews and subsequent deductive themes were structured around four key stages of the cancer trajectory: (1) illness onset, (2) diagnosis, (3) treatment, and (4) life beyond treatment. Inductive themes related to HRQoL were identified across these four stages (Table 2). In addition, general themes not specific to a particular stage were identified. These included (1) social support, (2) impact on others, (3) illness and the social self, (4) experiences with health care/information provision, and (5) positive reappraisal.

The thematic analysis section is structured according to the time-based themes and the general themes. Interview extracts are presented in quotation marks. Text in *italics* represents the patients’ direct words, while non-italicized quoted text reflects the interviewers’ notes. Patient characteristics appear in brackets (sex, age, and country—when the patient has metastatic cancer, this is also indicated; no indication means localized cancer). Themes and subthemes are shown in tables and highlighted in **bold** within the text.

#### 3.2.2. Time-Structured Themes

Table 2 (above) summarizes the thematic analysis of the cancer trajectory. Each theme and subtheme is described in the text.

#### 3.2.3. Illness Onset

For this period, patients’ accounts focused on **symptoms** and **trajectory**. The most common **symptoms** at onset were the presence of blood in the stool and changes in bowel habits, often characterized by alternating constipation and diarrhoea. At this stage, most patients did not experience pain, and only a few reported feeling a lump or abnormal growth formation. One patient vividly described this sensation: *“I felt like a chicken with an egg inside me that just couldn’t come out. I could clearly feel that something was there.”* (Female, 53, The Netherlands).

For some patients, these symptoms overlapped with those associated with pre-existing conditions, such as Crohn’s disease (one patient), haemorrhoids (three patients), and menopause (one patient), and thus lowered the suspicion of cancer. Two patients reported having no symptoms at all, discovering the tumour incidentally during a routine colonoscopy or “by chance” during follow-up for a liver transplant.

**Trajectory** encompassed the **actions taken** by patients to investigate symptoms. Due to the non-specific nature of most complaints (e.g., changes in bowel habits), patients expressed uncertainty about which medical specialty to consult. As one patient explained: *“I almost felt bad for visiting the doctor so often with vague complaints.”* (Female, 50 years old, The Netherlands).

The trajectory also involved **frustration of not being taken seriously** by healthcare professionals. One patient (Female, 53 years, UK) reported opting for a private consultation after feeling that the public health system dismissed her symptoms, attributing them to haemorrhoids.

### 3.3. Diagnosis

When discussing the period surrounding their cancer diagnosis, patients described experiences involving **symptoms** (progressive worsening), **trajectory** (diagnostic procedures, time to establish diagnosis, and misdiagnosis), **emotional responses** (shock, anxiety, fear, and despair), and **coping strategies** (practical actions, avoidance, and acceptance).

From the first signs of illness to the time of diagnosis, patients reported worsening **symptoms**, often accompanied by severe pain, as explained in the following extracts:

“*By that time* [diagnosis], *I couldn’t even sit anymore due to the pain, and I could feel the tumour from the outside*.” (Female, 53 years, The Netherlands).

“Using the bathroom became so unpleasant that she stopped wanting to eat and drink, which she found unbearable.” (Female, 50 years, The Netherlands).

The **trajectory** during diagnosis referred to decisions made by healthcare professionals to investigate patients’ symptoms. Most patients underwent colonoscopy, which was frequently described as “*awful and distressing*” (Female, 75 years, UK). Other **diagnostic procedures** included stool tests for parasites, ultrasound, and MRI scans.

Many patients (*n* = 5) initially received an **incorrect diagnosis**, including haemorrhoids, colitis, parasitic infection, and pelvic floor collapse: “More doctors looked [at anal wound] and were 100% certain it was an infected haemorrhoid, which reassured her since so many doctors confirmed it.” (Female, 56 years, The Netherlands).

Following misdiagnosis, symptoms (particularly bleeding and severe pain) continued to worsen, prompting patients to return to their healthcare providers.

For some, the diagnostic process extended over several months and involved multiple examinations and referrals to different specialists (e.g., general practitioners, gynaecologists, proctologists, gastroenterologists, dermatologists), leading to stress and frustration:

“*The entire process took an incredibly long time: from my first visit to the GP at the end of April to my first treatment at the end of August*.” (Female, 53 years, The Netherlands).

“*Lots of referrals, started with the gastroenterologist, then the oncologist, another oncologist, etc.—this is frustrating*.” (Female, 58 years, The Netherlands).

Two patients from The Netherlands reported a relatively rapid diagnosis following blood in the stools or an anal lump, with approximately one month between the first consultation and cancer detection. However, most described that the waiting period as highly stressful and emotionally taxing: “*That period* [waiting for final diagnosis] *when you don’t exactly know what is going on, but you do know the disease is serious and rare, was one of the darkest times*.” (Female, 65 years, The Netherlands).

For most patients, the cancer diagnosis came as a profound shock: “*I was shocked, didn’t expect it to be cancer. It hit hard. I went through some tough lows*.” (Female, 58 years, The Netherlands). Shock was pronounced among patients whose diagnosis occurred by chance (e.g., during investigations for another illness), as well as among young individuals and those with young families or dependents. One patient expressed: “*The diagnosis was a shock for me. I have two disabled children and my family needs me.*” (Female, 49 years, Austria).

Other **emotions** included anxiety, stress, and fear, both for themselves and for loved ones. Concerns ranged from genetic predisposition to the ability to care for dependents: “*It was stressful for me at first because I had 2 children at home and had just built the house at the time and of course you start to worry* […].” (Male, 63 years, Austria).

In some cases, fear escalated into despair, with one patient disclosing suicidal thoughts: “*During that time, all I could do was cry*. […] *Sometimes I thought, ‘I’ll just jump in front of a train*.’” (Female, 53 years, The Netherlands).

Patients employed various **coping strategies** during this period. **Practical actions** included psychotherapy, hypnotherapy, and using distraction techniques. One patient talked about the importance of psychotherapy: “*Many patients need mental support, like a psychologist—it really helps. Without that, a lot of people keep everything inside, and that’s not sustainable*.” (Female, 53 years, The Netherlands).

Other strategies involved **avoidance** (e.g., limiting information intake) and **acceptance**:

“*I was offered a lot of information, but I did not want to know too much about the side effects [of treatment] because I had no other option and I really wanted to have the treatment*.” (Male, 63 years, Austria. Subtheme: avoidance). “*I* *don’t* *want to know too much about my prognosis, I just want to get the therapy over with.*” (Female, 49 years, Austria, Subtheme: avoidance).

“*Well, something is going to take me* […] *Emotionally I was okay because of my age, not going to live forever.*” (Male, 76 years, UK. Subtheme: acceptance).

### 3.4. Treatment

Analyses of the data revealed six treatment-related themes: **side effects**, **treatment routine**, **disruption to daily activities**, **social functioning**, **emotional functioning**, and **coping strategies** (including medication, support from others, positive lifestyle changes, and reframing perspective).

The most frequently reported **side effects** were skin complications (soreness, wounds, and burns), fatigue, nausea, bloating, bowel incontinence, bowel urgency, neuropathy, swelling, weakness, and severe pain, including painful urination and painful bowel movement. Several patients described the treatment as “brutal,” with some requiring hospitalization and/or morphine for pain management. One patient reflected on the impact of morphine: *“The morphine makes you feel like you’re not even human anymore. I watched videos just to pass the time.”* (Female, 53 years, The Netherlands). Burns in the treated area often resulted in painful urination and defecation: *“It felt like there were shards of glass in my stool, accompanied by heavy bleeding.”* (Male, 77 years, The Netherlands).

Patients highlighted the demanding nature of the **treatment routine**. Daily hospital visits, often at varying times, coupled with long waiting periods, were described as exhausting: *“I found the radiation treatments mentally very challenging. I had to go to the hospital every day, always at a different time. On top of that, there was often a lot of waiting. It felt like my life was on hold for seven weeks, and I became completely drained, both physically and mentally.”* (Male, 77 years, The Netherlands). Two patients mentioned the difficulties and discomfort of having to achieve a full bladder for radiation.

Due to the intensive side effects and demanding treatment routines, patients reported significant **disruption to their daily activities**, including hobbies and the need to carefully plan their day. Changes in routine were primarily attributed to hospitalization (in a few cases) and symptoms such as pain, soreness, leakage of mucus, fatigue, bowel urgency, and bowel incontinence. Reported restrictions included the inability to work, cycle, perform household tasks, shop, take long walks, climb stairs, and the need to carry spare clothes and limit activities to locations near a toilet. A few patients described severe pain that restricted walking, sitting, and driving. Patient expressed the extent of these limitations:

“*Can’t do daily work. I can’t go out. I can’t go shopping. I can’t travel.*” (Female, 65 years, Turkey).

“*I now have to go to the loo very quickly as soon as I feel the urge, which bothers me as it restricts my everyday life.*” (Female, 49 years, Austria).

“*The last few weeks were truly awful, and my physical condition deteriorated significantly, even though I am normally very fit.*” (Male, 77 years, The Netherlands).

The emotional toll was significant, affecting **emotional functioning**. One account noted: “The patient felt she could no longer cope and described this period as truly awful.” (Female, 50 years, The Netherlands). Many patients reported insomnia and difficulty concentrating, noting that even simple activities such as reading became challenging.

**Social functioning** was similarly affected. As one patient explained: *“I’m currently concentrating on my therapy and my family… I am seeing less of my friends at the moment.”* (Female, 49 years, Austria). Patients often paused social activities during treatment, primarily due to symptoms and side effects, the treatment schedule, and, for some, a desire to avoid disclosing their diagnosis.

Patients employed various **coping strategies** to manage distress and pain, including medication (for pain, nausea, and insomnia) and support from others (family and friends): *“For my treatment, friends gave me 36 little bags—I could open one for each day of treatment. I’ve received a lot of support from everyone.”* (Female, 58 years, The Netherlands).

Some patients coped by **reframing their perspective**. This involved comparing their experience to others’ more severe cases: “Because others had shared such extreme stories about radiation, she felt her own experience wasn’t as bad as expected.*”* (Female, 56 years, The Netherlands). Others focused on positive aspects, such as feeling cared for by healthcare professionals or the relief of starting treatment: *“I felt so well cared for and treated like a person.”* (Female, 51 years, UK). 

When the patient started treatment, it was a relief: “*Now we’re finally doing something about it*.” (Female, 50 years, The Netherlands). **Positive lifestyle changes** were referred to during or in preparation for treatment, including smoking cessation, alcohol abstinence, healthy eating, and physical activity.

### 3.5. Life Beyond Treatment

Analyses of this period revealed four themes: **challenges** (emotional, physical, role-function limitations, disruption to daily activities, and sexual dysfunction), **changed life perspective**, **coping and recovery strategies** (practical actions, gratitude, and trauma growth), and **return to normality**.

Many patients described the post-treatment period as the most **challenging** phase: *“When you’re done with treatment, that’s when the real blow comes, and you fall into a hole.”* (Female, 56 years, The Netherlands).

The intensity of the treatment schedule seemed to leave little time for reflection during therapy, as one patient explained: *“I didn’t have much time to think about* [cancer] *after the diagnosis because I had so many appointments and I’m currently undergoing treatment, so I can’t worry too much.”* (Female, 62 years, Austria). After treatment ended, many struggled with uncertainty about the future, as observed in the following field note: “After the treatments ended, she wrestled with the question, ‘What now?’—realizing the journey was far from over. This uncertainty continues to weigh heavily on her and remains a major focus of her thoughts.” (Female, 50 years, The Netherlands).

**Emotional distress** was common, particularly anxiety about the future and fear of recurrence, which compromised willingness and ability to make plans: “The patient states that she is not depressed; she can still enjoy life, but she doesn’t feel carefree. She increasingly finds herself questioning the purpose of certain things. For example, she wonders why she should still make a dentist appointment if she might not be around much longer or why put so much energy into her job if she doesn’t have much time left.” (Female, 50 years, The Netherlands).

Fear also extended to treatment consequences: “*I’m also concerned about the potential long-term damage to organs that were affected by the radiation. And, of course, the question always remains whether the cancer will return.*” (Female, 53 years, The Netherlands).

Distress and fear were particularly pronounced among patients with small children and children with disabilities (two cases): “*The whole situation is very stressful for me because of my children* [both of them have a disability], *I’m worried about them*.” (Female, 49 years, Austria).

**Physical challenges** included persistent symptoms and side effects, most notably bowel incontinence and urgency, which restricted daily and social activities: “*The only thing I really need to consider now is my bowel movements. For example, taking a long flight is challenging, even though we used to travel a lot and would love to do so again. I simply have no control over when I need to go to the bathroom*.” (Male, 77 years, The Netherlands). Loss of bowel control significantly **disrupted patients’ ability to plan and engage in daily activities**, which often had to be adapted to ensure proximity to a toilet. A 50-year-old female patient from The Netherlands shared an emotional account of a “humiliating incident” involving bowel incontinence at a supermarket: a situation she still finds difficult to discuss and fears might happen again. As noted in the field report: “She is determined never to experience a situation like the one in the supermarket with her son again, as she remains deeply embarrassed by it.” Several other patients reported still feeling restricted in activities such as traveling, shopping, or simply going out due to bowel incontinence.

Persistent fatigue, pain, and soreness in the radiated area were also reported, often **compromising role functioning**, which comprised patients’ ability to function as before treatment. This pattern was well noted in the following: “She feels she can’t manage everything anymore—being a mother, a partner, working, maintaining the garden, and taking care of the dog and cat. This is very frustrating for her.” (Female, 50 years, The Netherlands). A few patients reported that decreased role functioning was compounded by other conditions (e.g., Crohn’s disease, heart failure, arthritis, hip replacement).

Patients also reported a loss of confidence in their bodies and the need to reduce the intensity of certain activities. As one participant explained: “*Being an athlete used to give me a lot of confidence in my body, but now that confidence is gone. I no longer trust my own body, and because of that, I avoid extreme activities*.” (Female, 53 years, The Netherlands).

**Sexual dysfunction** was mentioned by five patients, often accompanied by frustration and/or guilt:

“*My sexual functioning was severely impacted, and that is the aspect that bothers me the most today. I have been considering consulting a urologist to find solutions to this problem*.” (Male, 54 years, Brazil).

“Intimacy is an issue as they [patient and partner] are still interested in sex, but it is painful and difficult. Her husband understands but she feels guilty.” (Female, 51 years, UK, metastatic).

“The patient began talking about sexuality, mentioning that she finds it difficult to feel like a woman. She also brought this up with her doctor and is thinking about consulting a sexologist.” (Female, 65 years, The Netherlands).

For most other patients, sexual changes were not referred to as problematic, either due to pre-existing low sexual activity or loss of interest since diagnosis. Some brief responses suggested the possible avoidance of discussing sexual health.

Patients described a **shift in life perspective** following cancer treatment. For some, this was negative, rooted in prior traumatic experiences: “*I’ve had a very tough past with cancer. I lost both my mother and grandmother to cancer, very close to each other, and I witnessed it all up close—it was devastating. For that reason, I chose not to have children, so they wouldn’t have to experience the intensity of such an illness and the impact of death*.” (Female, 53 years, The Netherlands).

As **coping and recovery strategies**, patients employed practical actions such as planning trips, engaging in hobbies (reading and gardening), and seeking professional support (e.g., Chinese medicine, massage therapy, pelvic floor therapy). Coping mechanisms also included gratitude and trauma growth:

“I *think I can be glad that I was treated here in Austria*.” (Male, 63 years, Austria). “*I’m not the same as I used to be, but I’ve found a different path. I don’t know how much time I have left, so I just buy that concert ticket, which I would have doubted about before. I can still enjoy things, even though I’m sick*.” (Female, 53 years, The Netherlands).

Despite the pronounced challenges, three patients mentioned a **return to “normal life**” now that several years had passed since the end of treatment. As one patient noted: “*I no longer have any problems at all. I have completely normal bowel movements and fortunately am not incontinent and have no other itching or similar problems in the area. There are a few bread veins on my skin but that doesn’t bother me. I have been working 100% again for a few years now*.” (Male, 63 years, Austria).

#### 3.5.1. General Themes

Patients discussed four themes not necessarily related to one period in their illness trajectory: **social support** (support offered and strengthened relationships), **impact on others** (emotional reactions), **illness and the social self** (disclosure to others, stigma, embarrassment, social identity, social functioning, and social comparison), **experiences with health care,** and **positive reappraisal**. These themes and subthemes are summarized in Table 3 and described below.

#### 3.5.2. Social Support

Patients generally described family members and friends as highly **supportive** and caring throughout the illness trajectory, as one patient expressed: “*I’ve also received immense support from friends, family, and especially my wife. She has truly been my caregiver, and she has done an incredible job.*” (Male, 77 years, The Netherlands). In some cases, the experience was perceived as **strengthening relationships**:

“Interviewer: Have relationships with family/friends changed? Patient: ‘*They are more stable and you can see which friends you can rely on*.’” (Female, 66 years, Austria). 

“*Our relationship* [with partner] *has become stronger because, through everything we’ve been through, we’ve been very dependent on each other*.” (Male, 77 years, The Netherlands).

#### 3.5.3. Impact on Others

Despite the support received, several accounts highlighted the **emotional** strain experienced by family members, which sometimes led patients to conceal their own feelings to protect loved ones:

“*It was very hard for my partner. He didn’t know, as is understandable, how to deal with it. When I had to cry, he cried too. To avoid making him sad, I shut myself off and went off alone to cry. The same goes for my brother; when I cry in front of him, he can’t handle it either*.” (Female, 53 years, The Netherlands).

“During this time, she often thought about the impact on her family and tried to shield them as much as possible, relying on friends for support instead.” (Female, 50 years, The Netherlands).

The cancer diagnosis was described as “devastating,” particularly for families who had previously lost relatives to cancer: “Telling her parents was so awful because they had already lost her sister (to cervical cancer—HPV-related).” (Female, 51 years, UK).

In a few cases, family members (most often husbands) responded with avoidance: “*My husband is very stressed and very worried, I don’t think he can cope with the whole situation on his own, but he doesn’t want to talk about it*.” (Female, 49 years, Austria).

#### 3.5.4. Illness and the Social Self

Patients’ accounts varied considerably regarding **disclosure** of their cancer diagnosis. Only one patient reported openly sharing her diagnosis as a deliberate effort to reduce **stigma**: “*I feel there’s still a taboo around anal cancer—I’ve consciously shared this openly with everyone*.” (Female, 58 years, The Netherlands).

Most patients, however, preferred not to disclose their diagnosis beyond close family members. Common reasons included fear of being perceived or treated differently and concerns about their **social identity** being reduced to “that person with cancer.” For some, nondisclosure also served as a coping strategy, helping them maintain a sense of normalcy: “*Most people don’t know that I’m sick (like the people at the gym, for example), so they don’t treat me any differently. It helps me to forget about it for a while, put the illness out of my mind, and feel like I did before*.” (Female, 56 years, The Netherlands).

One patient expressed missing opportunities to connect with others who shared similar experiences: “*What I do miss is having people who are going through the same thing, with whom I can talk about what I’m experiencing*.” (Female, 53 years, The Netherlands). Shared experiences appeared to play an important role in providing social support and fostering positive **social comparison**, for example, by helping patients reframe their own situation in light of others’ more challenging experiences.

Two patients reported feeling particularly **embarrassed** and uncomfortable discussing anal cancer. One disclosed that she told most people her cancer was colorectal, while another emphasized privacy concerns related to treatment regimen and his strong decision not to reveal his diagnosis: “*I’m seeing fewer people around me at the moment because I want to keep my illness a secret*. […] *I find it very annoying that I have to come for radiotherapy every day. I also think that more should be done for data protection and privacy, I don’t want other people to see me here*.” (Male, 56 years, Austria).

The decision to keep their diagnosis private negatively affected **social functioning**. Others reported withdrawing from social activities due to fatigue and reduced energy. One interviewer explained: “The patient does fewer social activities. She goes to bed at 9 PM, which means she misses out on certain things, including social interactions at home. […] The patient says that people around her have noticed that she has changed.” (Female, 50, The Netherlands). Disruption in social functioning was not always perceived as troubling, as noted in one interview: “She is not so bothered to go out socially but is not concerned by this.” (Female, 75 years, UK).

#### 3.5.5. Experiences with Health Care and Information Received

Of the 21 interviews, five (conducted in Austria) included direct questions about patients’ experiences with the health care system, whereas the remaining interviews focused only on HRQoL issues. In both cases, however, most participants spontaneously shared their experiences with health care institutions and professionals, as well as the information they received.

Most patients reported **positive experiences** with health care, highlighting effective communication with professionals, logistical support for on-site treatment, and welcoming facilities:

“*When a hospital is small-scale, like the one in* [hospital’s name] *where I had my radiation treatments, it feels like coming home. There was a calm atmosphere, which was incredibly comforting*.” (Male, 77 years, The Netherlands). 

“*I am very satisfied with the doctor’s communication and the information I have received. The doctor took a lot of time for me and everyone here on the ward is very nice to me*.” (Female, 57 years, Austria).

Negative experiences were uncommon and primarily related to delays and inaccuracies in the initial diagnostic process: *“Later, my GP apologized for not having detected the tumour during the initial consultations. I found this very difficult to process*.” (Female, 53 years, The Netherlands). There were also a few accounts of receiving **incorrect information**, which caused significant distress: “At the point of the colonoscopy she was very upset because the clinician told her it was cancer (before further tests had been carried out) and that she would have a stoma and it would not be reversible. This caused immense distress. The patient recalled feeling ‘almost suicidal.’” (Female, 74 years, UK).

Participants also offered **recommendations** for improving care and communication. One patient suggested providing more detailed information about treatment side effects (Male, 54 years, Brazil). Another emphasized the need for better emotional support after treatment: *“It would be nice if there were more tips on what you can do after treatment when you fall into that hole; you have to figure this all out on your own.”* (Female, 58 years, The Netherlands).

#### 3.5.6. Positive Reappraisal

**Positive reappraisal** was identified as an interpretive theme rather than being explicitly labelled by patients. Many narratives reflected positive aspects throughout the cancer trajectory, including gratitude (toward health care providers and for the body’s ability to cope and heal) and strengthened relationships with loved ones:

“She is very grateful for her body and how well it has healed. While she takes good care of her body (exercise, healthy eating), she also feels lucky with how well her body has responded.” (Female, 56 years, The Netherlands). “*I feel very supported by friends and family—my perspective has changed in a positive way*.” (Female, 58 years, The Netherlands). “It made her feel lucky to receive treatment and to have a positive outcome.” (Female, 75 years, UK).

#### 3.5.7. Questionnaires

No floor or ceiling effects were observed for any EORTC QLQ-C30 items, indicating that the questionnaire was sufficiently sensitive to differentiate between participants across the full range of responses. The questionnaire data supported patients’ accounts of their prominent physical symptoms. The EORTC QLQ-C30 question about pain had the highest mean score (71.4%; mean: 3.62; SD = 0.74), aligning with the severe pain patients described throughout their cancer trajectory (mostly during diagnosis and treatment). In the EORTC QLQ-ANL27, the highest mean score was in question 37, “soreness in the areas that have been treated”, where 71.4% of patients responded, “very relevant” (mean 3.71; SD = 0.46). This finding mirrors the interview data, where soreness was consistently described as a persistent challenge from treatment onwards. Full scores and means can be found in Table 4 and Table 5.

The most relevant question for patients was in the EORTC QLQ-ANL27 and was related to soreness in the areas that had been treated (61.9%), followed by leakage of stools and pain/discomfort around their anal opening (both 57.1%). In the EORTC QLQ-C30, the pain question was the most frequently rated among the top 10 most relevant questions, selected by 48% of patients. Table 6 and Table 7 identify questions from each questionnaire that were selected as part of the top 10 for relevance by more than 25% patients.

The high relevance attributed to pain, soreness, and stool leakage reflects the major physical difficulties reported in interviews, where severe pain and bowel incontinence were described as significantly disrupting daily roles and social functioning.

## 4. Discussion

This mixed-methods study provides an in-depth account of how anal cancer and its treatment affect patients’ HRQoL across the illness trajectory. By combining patient interviews with relevance ratings of generic and tumour-specific HRQoL questionnaires, this study highlights both the adequacy of existing instruments in capturing physical symptom burden and their limitations in addressing the social, emotional, and stigma-related dimensions of living with anal cancer. Given the scarcity of research on anal cancer, this study provides valuable insights into the emotional burden, stigma, diagnostic delays, and post-treatment vulnerability experienced by anal cancer patients, reflecting patient voices that have been minimally represented in prior research.

Beyond experiencing challenges due to its rarity, patients with anal cancer also deal with acute and long-lasting effects that severely impact their HRQoL [31]. Patients’ accounts of problems relating to bowel, urinary, and sexual dysfunction support what is known in the literature [6] and what is covered by the tumour-specific questionnaire EORTC QLQ-ANL27 [14]. Although the profound impact of the intense radiation applied to the functionality of the organs treated is widely reported, the emotional consequences of it, alongside receiving and dealing with the diagnosis itself, and treatment options remain understudied.

A recurrent theme from the interviews was the prolonged and often frustrating diagnostic process. Many patients reported initial misdiagnoses frequently as haemorrhoids, which delayed appropriate care and were perceived to exacerbate symptoms, including severe pain that, in some cases, required opioid management. Previous studies have reported similar experiences of incorrect diagnosis in patients with other rare cancers [32,33,34], which in turn led to a negative impact on HRQoL [32]. To address this issue and streamline the diagnosis process, initiatives have been created to advise healthcare systems. One example is The Netherlands Cancer Institute’s campaign, which aims to break the silence around rare and stigmatised cancers and try to improve patients’ awareness of symptoms that can be noticed during a toilet visit; this campaign has been created to advise healthcare systems [35].

Stigma and embarrassment were also prominent themes linked to both the uncommon nature of the disease and its intimate symptoms and side effects. As noted in the analysis of interviews with anal cancer patients in Denmark, “the real problem is the word anal and not cancer” [36] (pg. 1218). This stigma extends to symptoms, particularly bowel urgency and incontinence, which are perceived as “a humiliating affair” [36]. Consistent with previous research, bowel incontinence was identified as one of the most significant side effects of anal cancer, impacting quality of life [36,37]; it is an issue that starts at onset, worsens during treatment, and persists as a chronic problem for anal cancer survivors. In our study, patients described how the lack of bowel control restricted daily functioning and led to humiliating incidents, echoing findings among Danish anal cancer survivors [36]. Similarly to other so-called “taboo” cancers, patients with penile cancers have reported social anxiety (e.g., when using public restrooms) and changes in their quality of life [38]. To help decrease the stigma and embarrassment, healthcare institutions and providers could facilitate meetings between patients, where stories could be shared and more peer support could be fostered. This approach was supported by a patient in the present study who mentioned a motivation to openly disclose her diagnosis to challenge taboos. The literature on penile cancer and sarcoma patients has shown the benefit and desire of communicating with individuals with the same condition [39,40]. Further, national campaigns can also aid in the loss of taboo when speaking about anal cancer.

The tendency of some patients to suppress their suffering to protect loved ones has been documented in other cancer contexts [41] and described as “double suffering” [42]. Emotional suppression for the sake of others was also observed in an earlier Danish study of anal cancer survivors, which linked this behaviour to the concept of modesty [36]. In this context, modesty refers to how patients navigate interactions with healthcare professionals, family, and social networks while balancing demands for discretion. This dynamic reflects a broader lack of openness surrounding anal cancer diagnoses and the associated stigma and suffering [36]. This finding underscores the importance of proactively assessing emotional distress rather than relying on spontaneous disclosure.

The theme of positive reappraisal was observed across several narratives and was associated with strong social support and positive interactions with healthcare providers. Positive reappraisal is a common coping strategy in the scientific literature on trauma [43]. This coping mechanism involves reframing adverse experiences in a more constructive light, which enhances resilience and psychological well-being [44]. Positive reappraisal has also been linked to post-traumatic growth, defined as positive psychological changes following major life crises [44]. Aspects of post-traumatic growth were apparent in some patients’ accounts in the present study, who described developing a renewed appreciation for life and granting themselves permission to pursue experiences they might previously have hesitated to embrace. While positive reappraisal appeared adaptive for some patients, it should not be interpreted as universal or expected, nor as a substitute for adequate psychosocial support.

Overall, patients expressed satisfaction with their healthcare experiences during treatment, often describing feelings of dignity associated with personalized care. It is important to note, However, that these positive experiences cannot be generalized to all healthcare systems. The fact that most participants in the present study were treated in large, well-equipped centres or university hospitals may have influenced the results. Nonetheless, the post-treatment phase was described as particularly challenging. Reduced contact with healthcare professionals left patients feeling abandoned and “on their own”, a common complaint in patients with rare cancers [45]. This sense of isolation echoes findings from Ueland et al., where cancer survivors reported diminished empathy and recognition of their struggles after treatment completion [41]. In our study, this isolation was compounded by persistent uncertainty regarding recurrence, concerns for loved ones’ health (due to genetic predisposition), and fears about long-term treatment effects. These findings point to a critical gap in transitional and survivorship care rather than dissatisfaction with treatment itself.

The current findings not only demonstrate the need for tailored psychosocial support and services that address the unique challenges experienced by patients with anal cancer but also highlights that a holistic approach is necessary when evaluating patients’ HRQoL. The current tools used to capture these patients’ experiences are relevant and specific, but an even more comprehensive strategy is needed. In practical terms, this could include systematically screening for psychosocial distress at key points along the care pathway, ensuring timely referral to psycho oncology services, and establishing clear communication pathways between oncology, mental health professionals, and supportive care teams to coordinate individualized care. By structuring follow-up pathways, continuity of care beyond active treatment also allows emotional burden and unmet psychosocial needs to be evaluated in the post-treatment phase. Further, by acknowledging the value of psychological support, one should advocate for its integration into routine care, and assistance should extend to family members, who often struggle to cope and may resort to unsupportive strategies such as avoidance or withdrawal.

### Strengths and Limitations

A key strength of this study is the inclusion of a considerable number of participants with an anal cancer diagnosis, representing diverse disease and treatment stages. This heterogeneity provides a broad range of perspectives from different countries, which is particularly valuable in under-researched areas by providing a wide range of viewpoints and leading to richer and more representative findings [46]. To our knowledge, this is the first in-depth study that investigates HRQoL in anal cancer, which adopts both qualitative and quantitative methods. Although the sex distribution in our sample is skewed toward females, this reflects the epidemiological pattern of anal cancer: Incidence among women has increased over the past four decades, largely due to its strong association with human papillomavirus (HPV). More than 90% of anal cancers diagnosed worldwide are linked to HPV, to which women appear to be particularly vulnerable [47].

While the strengths outlined above are substantial, it is important to acknowledge that the stigmatized nature of the anal cancer condition might have restricted patients’ willingness to disclose further details on their HRQoL, especially concerning sexual functioning. No cultural patterns were identified, possibly likely due to the limited representation of participants outside Europe (one from Brazil and one from Turkey). Although qualitative research demonstrates that the perspective of even a single participant can offer meaningful insights into cultural phenomena, the predominance of European participants in this study restricts the extent to which broader conclusions can be drawn about experiences in other regions [48]. In addition, the relatively small sample size and the use of convenience sampling further limit the transferability of the findings, as certain cultural contexts may be underrepresented or absent. These sampling characteristics may influence which experiences and viewpoints were more prominent in the analysis and, therefore, should be considered when interpreting the broader applicability of the results. Finally, considering that all EORTC QLQ-ANL27 items were presented to all participants, regardless of conditional instructions in the original questionnaires, results relating to relevance should be interpreted with caution for items 47–49 (regarding stoma) and 54–57 (regarding erection and vaginal issues).

## 5. Conclusions

Patients with anal cancer face multiple HRQoL challenges, including those common to the broader cancer experience and additional burdens linked to the rarity and stigma of this condition. The results highlight the usefulness and relevance of HRQoL questionnaires in assessing anal-specific physical symptoms that matter to patients. However, social and emotional aspects are not comprehensively covered. Experiences with health care were perceived as very positive, except at the time of diagnosis and once treatment ended. These findings highlight the need for healthcare professionals to recognize and address the unique challenges through specialized, well-coordinated care.

Patients’ narratives suggest that clear communication and individualized support can foster positive reappraisal and facilitate adaptive coping strategies. Furthermore, rehabilitation and post-treatment interventions should be implemented to help patients manage persistent distress, physical limitations, and feelings of isolation. Extending psychosocial and practical support beyond active treatment is essential to promote long-term well-being, quality of life, and dignity in survivorship.

## Figures and Tables

**Table 1:** Sociodemographic and clinical characteristics of patients.

I. Sociodemographic Characteristics	Patients (*n* = 21)	
	*n*	(%)
**Age**		
Mean (SD)	61.9 (SD 9.3)	
Range	49–77	
**Sex**		
Male	6	28.6
Female	15	71.4
**Civil status**		
Single	5	23.8
Partner	16	76.2
**Living situation**		
Alone	5	23.8
Not alone	16	76.2
**Highest level of education ^a^**		
Low	0	0
Medium	8	38.1
High	13	61.9
**Employment status ^b^**		
Not actively employed	14	66.7
Actively employed	7	33.3
**Country**		
The Netherlands	6	28.6
UK	7	33.3
Brazil	1	4.8
Austria	6	28.6
Turkey	1	4.8
**Travel time to hospital**		
<15 min	3	14.3
15–30 min	3	14.3
30–1 h	12	57.1
1–2 h	3	14.3
2+ h	0	0
**Travel distance to hospital**		
<10 km	5	23.8
10–25 km	3	14.3
25–50 km	7	33.3
50–100 km	6	28.6
100+ km	0	0
**II. Clinical characteristics**		
**Time since cancer diagnosis (months)**		
Mean (SD)	17.6 (SD 17.4)	
Range	1–70	
**Type of disease**		
Localised disease	19	90.5
Metastatic disease	2	9.5
**Presence of metastasis**		
No	19	90.5
Yes	2	9.5
**Number of metastases**		
0	19	90.5
1–3	1	4.8
4 or more	1	4.8
**Comorbidities ^c^**		
0	12	57.1
1	7	33.3
2 or more	2	9.5
**Treatment goal**		
Curative	19	90.5
Palliative	2	9.5
**Treatment modality**		
Chemotherapy ^d^	15	71.4
Radiotherapy ^e^	15	71.4
Surgery	3	14.3
Resection	1	4.8
Other ^f^	8	38.1
**ECOG Status ^g^**		
0	12	57.1
1	6	28.6
2	3	14.3

^a^ Low: No or primary school education; medium: secondary education, including high school; high: college or university degree. ^b^ Not actively employed: retired, sick leave, or unemployed; actively employed: full-time, part-time. ^c^ Heart failure, autoimmune disease (rheumatoid arthritis, SLE, Crohn’s disease), kidney disease, liver disease, depression, anxiety disorder, cirrhosis, osteoporosis, hypertension, liver transplant, hip-TEP right and left, nerve damage. ^d^ Included: clinical; day care treatment. ^e^ Included: local curative neoadjuvant; local curative adjuvant; local high-dose palliative, localized low-dose palliative, proton therapy. ^f^ Other: Chemoradiotherapy (*n* = 7), steroids (*n* = 1). ^g^ Eastern Cooperative Oncology Group Performance Status.

**Table 2:** Themes and subthemes related to the key stages of the cancer trajectory.

Illness Trajectory Stage	Theme	Subthemes
Illness onset	Symptoms	Common symptoms Overlapping symptoms
	Trajectory	Actions taken Frustration of not being taken seriously
Diagnosis	Symptoms	Progressive worsening
	Trajectory	Diagnostic procedures Time to establish diagnosis Misdiagnosis
	Emotional responses	Shock Stress Fear (for oneself and others) Despair
	Coping strategies	Practical actions Avoidance Acceptance
Treatment	Side effects	Common side effects
	Treatment routine	Challenges
	Disruption to daily activities	
	Emotional functioning	
	Social functioning	
	Coping strategies	Medication Reframing perspective Support from others Positive lifestyle changes
Life beyond treatment	Challenges	Emotional distress: uncertainty, fear Physical challenges Role function limitations Disruption to daily activities Sexual dysfunction
	Coping & recovery strategies	Gratitude Trauma growth Practical actions
	Changed life perspective	Post-trauma decisions
	Return to normality	

**Table 3:** General themes and subthemes on patients’ quality of life and health care experience.

Theme	Subtheme
Social support	Support offered Strengthened relationships
Impact on others	Emotional reactions
Illness and the social self	Disclosure to others Stigma Embarrassment Social identity Social comparison Social functioning
Experiences with health care/information provision	Positive experiences Negative at onset: incorrect information Recommendations
Positive reappraisal	Received health care Body’s ability to heal Strengthened relationships

**Table 4:** Participant relevance ratings of EORTC QLQ-C30 ^a^ questionnaire items.

	1 Not Relevant *n* (%)	2 Somewhat Relevant *n* (%)	3 Relevant *n* (%)	4 Very Relevant *n* (%)	Mean	SD	Cut-Off 1.5 (y/n)
Item 1: trouble doing strenuous activities, like carrying a heavy bag	7 (33.3)	5 (23.8)	4 (19)	5 (23.8)	2.33	1.20	Yes
Item 2: trouble taking a long walk	6 (28.6)	4 (19)	7 (33.3)	4 (19)	2.43	1.12	Yes
Item 3: trouble taking a short walk	11 (52.4)	4 (19)	3 (14.3)	3 (14.3)	1.90	1.14	Yes
Item 4: need to stay in bed or a chair during the day	9 (42.9)	6 (28.6)	2 (9.5)	4 (19)	2.05	1.16	Yes
Item 5: need help with eating, dressing, washing or using the toilet	12 (57.1)	6 (28.6)	1 (4.8)	2 (9.5)	1.67	0.97	Yes
Item 6: limited in doing either work or other daily activities	6 (28.6)	5 (23.8)	4 (19)	6 (28.6)	2.48	1.21	Yes
Item 7: limited in pursuing hobbies or other leisure time activities	6 (28.6)	6 (28.6)	7 (33.3)	2 (9.5)	2.24	1.00	Yes
Item 8: short of breath	10 (47.6)	5 (23.8)	4 (19)	2 (9.5)	1.90	1.04	Yes
Item 9: pain	1 (4.8)	0	5 (23.8)	15 (71.4)	3.62	0.74	Yes
Item 10: need to rest	2 (9.5)	5 (23.8)	5 (23.8)	7 (33.3)	2.90	1.00	Yes
Item 11: trouble sleeping	4 (19)	3 (14.3)	7 (33.3)	7 (33.3)	2.81	1.12	Yes
Item 12: felt weak	2 (9.5)	5 (23.8)	10 (47.6)	4 (19)	2.76	0.89	Yes
Item 13: lacked appetite	4 (19)	7 (33.3)	6 (28.6)	4 (19)	2.48	1.03	Yes
Item 14: nausea	8 (38.1)	3 (14.3)	7 (33.3)	3 (14.3)	2.24	1.14	Yes
Item 15: vomited	9 (42.9)	7 (33.3)	3 (14.3)	2 (9.5)	1.90	1.00	Yes
Item 16: constipation	5 (23.8)		7 (33.3)	9 (42.9)	2.95	1.20	Yes
Item 17: diarrhoea	1 (4.8)	1 (4.8)	9 (42.9)	10 (47.6)	3.33	0.80	Yes
Item 18: tiredness	1 (4.8)	5 (23.8)	5 (23.8)	10 (47.6)	3.14	0.96	Yes
Item 19: pain interfere with daily activities	3 (14.3)	3 (14.3)	6 (28.6)	9 (42.9)	3.00	1.10	Yes
Item 20: difficulty in concentrating on things, like reading a newspaper or watching television	5 (23.8)	9 (42.9)	3 (14.3)	4 (19)	2.29	1.06	Yes
Item 21: feel tense	5 (23.8)	6 (28.6)	4 (19)	6 (28.6)	2.52	1.17	Yes
Item 22: worry	5 (23.8)	6 (28.6)	2 (9.5)	8 (38.1)	2.62	1.24	Yes
Item 23: irritable	8 (38.1)	6 (28.6)	4 (19)	3 (14.3)	2.10	1.09	Yes
Item 24: depressed	6 (28.6)	6 (28.6)	4 (19)	5 (23.8)	2.38	1.16	Yes
Item 25: difficulty remembering things	10 (47.6)	5 (23.8)	2 (9.5)	4 (19)	2.00	1.18	Yes
Item 26: physical condition or medical treatment interfered with family life	5 (23.8)	6 (28.6)	6 (28.6)	4 (19)	2.43	1.08	Yes
Item 27: physical condition or medical treatment interfered with social activities	3 (14.3)	7 (33.3)	5 (23.8)	6 (28.6)	2.67	1.06	Yes
Item 28: physical condition or medical treatment caused financial difficulties	12 (57.1)	3 (14.3)	4 (19)	2 (9.5)	1.81	1.08	Yes
Item 29: rate overall health		1 (4.8)	13 (61.9)	7 (33.3)	3.29	0.56	Yes
Item 30: rate overall quality of life		2 (9.5)	12 (57.1)	7 (33.3)	3.24	0.62	Yes

^a^ EORTC QLQ-C30 refers to the core quality of life questionnaire of the European Organisation for Research and Treatment of Cancer.

**Table 5:** Participant relevance ratings of EORTC QLQ-ANL27 ^a^ questionnaire items.

	1 Not Relevant *n* (%)	2 Somewhat Relevant *n* (%)	3 Relevant *n* (%)	4 Very Relevant *n* (%)	Mean	SD	Cut-Off 1.5 (y/n)
Item 31: leakage of stools or mucus from anal opening (back passage)		3 (14.3)	6 (28.6)	12 (57.1)	3.43	0.75	Yes
Item 32: experienced frequent bowel movements	2 (9.5)	1 (4.8)	7 (33.3)	11 (52.4)	3.29	0.96	Yes
Item 33: bowel movements have been painful	2 (9.5)		5 (23.8)	14 (66.7)	3.48	0.93	Yes
Item 34: pain/discomfort around anal opening (back passage)	1 (4.8)	2 (9.5)	3 (14.3)	15 (71.4)	3.52	0.87	Yes
Item 35: pain while sitting	3 (14.3)	2 (9.5)	4 (19)	12 (57.1)	3.19	1.12	Yes
Item 36: uncomfortable in certain positions (e.g., lying down)	6 (28.6)	3 (14.3)	7 (33.3)	5 (23.8)	2.52	1.17	Yes
Item 37: soreness in the areas that have been treated			6 (28.6)	15 (71.4)	3.71	0.46	Yes
Item 38: itchy or irritated skin in the areas that have been treated	1 (4.8)	2 (9.5)	8 (38.1)	10 (47.6)	3.29	0.85	Yes
Item 39: need to urinate frequently	5 (23.8)	3 (14.3)	5 (23.8)	8 (38.1)	2.76	1.22	Yes
Item 40: swelling in legs or ankles	10 (47.6)	7 (33.3)	3 (14.3)	1 (4.8)	1.76	0.89	Yes
Item 41: problems going out of the house because needed to be close to a toilet	5 (23.8)	3 (14.3)	2 (9.5)	11 (52.4)	2.90	1.30	Yes
Item 42: need to clean themselves more often	6 (28.6)	5 (23.8)	6 (28.6)	4 (19)	2.38	1.12	Yes
Item 43: problems planning activities in advance (e.g., meeting friends)	7 (33.3)	4 (19)	4 (19)	6 (28.6)	2.43	1.25	Yes
Item 44: problems with gas (flatulence)	3 (14.3)	6 (28.6)	6 (28.6)	6 (28.6)	2.71	1.06	Yes
Item 45: urge to move bowels. hurry to get to the toilet	4 (19)	2 (9.5)	5 (23.8)	10 (47.6)	3.00	1.18	Yes
Item 46: feeling of being unable to completely empty bowels	4 (19)	6 (28.6)	4 (19)	7 (33.3)	2.67	1.15	Yes
Item 47: sore skin around stoma	17 (81)		2 (9.5)	1 (4.8)	1.35	0.88	No
Item 48: leakage of stools from stoma bag	18 (85.7)		1 (4.8)	1 (4.8)	1.25	0.79	No
Item 49: unintentional release of gas/flatulence from stoma bag	16 (76.2)		2 (9.5)	2 (9.5)	1.50	1.05	Yes
Item 50: sexually active	7 (33.3)	4 (19)	5 (23.8)	5 (23.8)	2.38	1.20	Yes
Item 51: extent of interested in sex	7 (33.3)	5 (23.8)	5 (23.8)	4 (19)	2.29	1.15	Yes
Item 52: disease or treatment affected sex life (for the worse)	7 (33.3)	5 (23.8)	6 (28.6)	3 (14.3)	2.24	1.09	Yes
Item 53: pain during intercourse	10 (47.6)	4 (19)	3 (14.3)	4 (19)	2.05	1.20	Yes
Item 54: difficulty getting or maintaining an erection	16 (76.2)	2 (9.5)	1 (4.8)	2 (9.5)	1.48	0.98	No
Item 55: vagina felt dry	8 (38.1)	5 (23.8)	2 (9.5)	6 (28.6)	2.29	1.27	Yes
Item 56: vagina felt narrow/tight	9 (42.9)	5 (23.8)	3 (14.3)	4 (19)	2.10	1.18	Yes
Item 57: pain in vagina	10 (47.6)	5 (23.8)	3 (14.3)	3 (14.3)	1.95	1.12	Yes

^a^ EORTC QLQ-ANL27 refers to the anal cancer questionnaire of the European Organisation for Research and Treatment of Cancer.

**Table 6:** Ten most relevant: those items reported by patients in the EORTC QLQ-C30 ^a^ questionnaire.

EORTC Questionnaire. Item No.	Description	Number of Patients (%)
EORTC QLQ-C30: Item 9	How relevant is to be asked if you had pain?	10 (47.6%)
EORTC QLQ-C30: Item 16	How relevant is to be asked if you have been constipated?	8 (38.1%)
EORTC QLQ-C30: Item 17	How relevant is to be asked if you had diarrhoea?	8 (38.1%)
EORTC QLQ-C30: Item 18	How relevant is to be asked if you are tired?	7 (33.3%)

^a^ EORTC QLQ-C30 refers to the core quality of life questionnaire of the European Organisation for Research and Treatment of Cancer.

**Table 7:** Ten most relevant: those items reported by patients in the EORTC QLQ-ANL27 ^a^.

EORTC Questionnaire. Item No.	Description	Number of Patients (%)
EORTC QLQ-ANL27: Item 37	How relevant is to be asked if you had soreness in the areas that have been treated?	13 (61.9%)
EORTC QLQ-ANL27: Item 31	How relevant is to be asked if you had leakage of stools or mucus from your anal opening (back passage)?	12 (57.1%)
EORTC QLQ-ANL27: Item 34	How relevant is to be asked if you had pain/discomfort around your anal opening (back passage)?	12 (57.1%)
EORTC QLQ-ANL27: Item 33	How relevant is to be asked if your bowel movements have been painful?	10 (47.6%)
EORTC QLQ-ANL27: Item 35	How relevant is to be asked if you had pain while sitting?	9 (42.9%)
EORTC QLQ-ANL27: Item 32	How relevant is to be asked if you have experienced frequent bowel movements?	8 (38.1%)
EORTC QLQ-ANL27: Item 45	How relevant is to be asked if you felt the urge to move your bowels. did you have to hurry to get to the toilet?	8 (38.1%)
EORTC QLQ-ANL27: Item 38	How relevant is to be asked if you had itchy or irritated skin in the areas that have been treated?	6 (28.6%)
EORTC QLQ-ANL27: Item 39	How relevant is to be asked if you had to urinate frequently?	6 (28.6%)

^a^ EORTC QLQ-ANL27 refers to the anal cancer questionnaire of the European Organisation for Research and Treatment of Cancer.

## Data Availability

Due to the sensitive nature of the questions asked in this study, patients were assured that raw data would remain confidential without any information to identify them.
